# Genetic and Phenotypic Associations of the Polygenic Score of Delay Discounting and Life History Traits

**DOI:** 10.1002/ajhb.70192

**Published:** 2026-01-07

**Authors:** Martin Fieder, Susanne Huber

**Affiliations:** ^1^ Department of Evolutionary Anthropology University of Vienna Vienna Austria

## Abstract

**Objectives:**

Delay discounting reflects individual differences in future orientation and impulsivity and may relate to life‐history strategies. Because delay discounting has a substantial genetic basis, we investigated whether the polygenic score (PGS) for delay discounting is associated with genetic predispositions for key life‐history traits—education, age at first birth, and number of children—and whether these relationships are reflected phenotypically.

**Methods:**

We used data from the Wisconsin Longitudinal Study, including 2713 men and 2980 women of European ancestry with available genetic data. Linear regressions examined associations between the delay‐discounting PGS and PGSs for educational attainment, age at first birth, and number of children. Parallel models assessed phenotypic associations with years of postsecondary education, age at first birth, and number of children. All models controlled for birth year and the first 10 genomic principal components.

**Results:**

In both sexes, the delay‐discounting PGS was strongly negatively associated with the PGSs for educational attainment and age at first birth, and positively associated with the PGS for number of children. Phenotypic associations were directionally consistent but substantially smaller: higher delay‐discounting PGSs predicted fewer years of education, earlier first birth, and (marginally) more children. Explained variance ranged from approximately 4%–5% for education to 1%–2% for reproductive traits.

**Conclusion:**

Genetic and phenotypic associations between delay discounting, education, and reproductive timing align with predictions from fast–slow life‐history theory. These findings suggest that behavioral tendencies related to impulsivity and future orientation share molecular genetic foundations with key life‐history traits while leaving substantial scope for environmental influences.

## 1 Introduction

Life history theory predicts that individuals optimize the allocation of their available resources to maximize fitness according to external conditions; accordingly, they experience a tradeoff between energy allocation for growth and survival versus reproduction as well as that between the quantity and quality of offspring, for example the investment in each descendant (Mace 2000; Mace 2007). Such a relationship has been observed, for example, in pre‐industrial societies where mothers with high fertility rates experienced higher infant mortality (Mace 2007; Strassmann and Gillespie 2002). Consequently, reducing the number of offspring while investing more in each individual child may be a strategy to increase overall fitness (Hill and Kaplan 1999; Penn and Smith 2007). This approach is especially relevant for women, given their substantial reproductive investment during pregnancy and breastfeeding. Hence, maximizing fitness “here and now” or planning for the future resource allocation and reproduction. Anyhow, it has been frequently criticized that the term life history is somehow blurred and differs between psychology and evolutionary biology. Nettle and Frankenhuis (2020) examined the divergence between life‐history theory in psychology (LHT‐P) and evolutionary biology (LHT‐E). LHT‐E focuses more on evolutionary trade‐offs that shape species‐level traits through natural selection, often using explicit mathematical models to predict how organisms allocate energy to survival, growth, and reproduction, whereas LHT‐P is more concerned with measuring individual differences along a fast‐slow continuum, reflecting how personal environments influence psychological and behavioral traits.

Life history theory in psychology (LHT‐P) usually emphasizes the role of environmental conditions—such as childhood adversity, unpredictability, or resource scarcity—in shaping individual fast–slow life history strategies. In contrast, evolutionary biology (LHT‐E) highlights inherited variation and trade‐offs that evolve through selection. These two approaches generate similar predictions but differ in their emphasis on environmental versus biological origins. The present study may contribute to bridging perspectives by investigating whether genetic predispositions related to delay discounting align with traits important to life‐history. Finding such genetic associations would suggest that individual differences relevant to fast–slow strategies may arise not only from environmental calibration but also from inherited tendencies.

Education is considered from evolutionary life history theory as an especially important trait in humans indicating a “slow life history”. Accordingly, education has been conceptualized within the framework of evolutionary life history as an investment in “embodied capital”—that is, in skills, knowledge, cognitive capacities, and social competencies that pay off later in life by enhancing reproductive success and survival. In humans, education is seen as part of a broader suite of adaptations that make up the “human adaptive complex”. As compared to other primates, humans exhibit unusually delayed reproduction, extended childhoods, and large brains. As a result, humans depend on intensive investment in education and skill acquisition during childhood and adolescence (Del Giudice et al. 2015). Thus, children not only rely on care and food, but also on the transmission of cultural knowledge by education (Kaplan et al. 2000).

Furthermore, human reproductive patterns and life stages form a key part of evolutionary approaches to life history. Humans have distinctive traits, including large, dependent babies, long childhoods, early menopause, and significant parental investment. These features involve trade‐offs between the number of children, the onset of reproduction, survival, and the success of future offspring. Consequently, the number of children and the age at which reproduction begins are key variables in human evolutionary life history (Mace 2000).

The concept of delay discounting is aligned with life‐history–relevant decision‐making because it captures individual variation in future orientation versus impulse control—traits that may influence life‐history outcomes. Thus, frequently tested “delay discounting” could bring new insights to the discussion on life history as delay discounting has a substantial genetic basis. Delay discounting is a fundamental aspect of impulsivity linked to various psychiatric disorders such as attention‐deficit/hyperactivity disorder (ADHD) (Jackson and MacKillop 2016), substance use disorders, obesity, and major depressive disorder (Amlung et al. 2017; McClelland et al. 2016). Accordingly, delay discounting refers to the tendency to devalue rewards as the delay to their receipt increases, reflecting a fundamental aspect of impulse control and thus also to some extent essential aspects of life history theory (Bari and Robbins 2013; Hamilton et al. 2015).

Twin studies indicate a substantial heritable component of delay discounting, ranging from 46% to 62% (Anokhin et al. 2015). Recently the genetic basis of delay discounting has been elucidated by Sanchez‐Roige et al. (2018) by utilizing a genome‐wide association study (GWAS) approach with 23 217 participants of European ancestry, recruited through the 23 and Me research platform. Participants completed the Monetary Choice Questionnaire (MCQ) to assess delay discounting preferences, which specifically measured the tendency to prefer smaller immediate financial rewards over larger delayed ones. The analysis tested SNP variants (single nucleotide polymorphism) for an association with delay discounting using a linear regression model, controlling for age, sex, and population stratification; identifying the SNP variant rs6528024, a single nucleotide polymorphism (SNP) within the GPM6B gene on the X chromosome, as the most significant genetic variant associated with delay discounting (*p* = 2.40 × 10^−8^). This gene is linked to serotonin transporter regulation, which is known to influence impulsive behavior. The GWA heritability of delay discounting was estimated to be around 12%, albeit—as usually—lower compared to twin studies, known as “missing heritability,” but suggesting that a significant portion of the trait is genetically determined. The study also found notable genetic correlations, including positive associations with ADHD, major depressive disorder, neuroticism, and smoking, while negative correlations were observed with cognitive traits like educational attainment and childhood IQ, indicating that the genetic basis of delay discounting overlaps with both psychiatric conditions and cognitive abilities.

To analyze a possible link between delay discounting and life history related traits, the present study aims to investigate on basis of the Wisconsin Longitudinal study whether the genetic predisposition (polygenic score) of delay discounting is associated with:

- The genetic predisposition in terms of the polygenic scores for education, age at first birth, number of children, that is polygenic scores that predispose important life history traits, and
- Life history phenotypes in terms of the number of children and age at onset of reproduction, as well as the highest level of education

Correlations or regressions among polygenic scores (PGS) are not direct estimates of biological pleiotropy or genetic correlation, as their magnitude can be inflated by shared discovery GWAS samples, GWAS sample size, error, and population structure (Kullo et al. 2022). Throughout this paper, we therefore interpret PGS–PGS associations only as indicators of directional genetic effects, not as quantitative estimates of shared genetic architecture.

## 2 Methods

The Wisconsin Longitudinal Study (WLS) is a long‐term panel study of a random sample of individuals who graduated from Wisconsin high schools in 1957, along with their siblings. The original WLS panel included 10 317 members of the 1957 graduating class. For this analysis, only individuals were included for whom genetic data were available and who were unrelated, resulting in a sample of 2713 white men and 2980 white women born between 1937 and 1940. We restricted the sample to white individuals to avoid issues with population stratification in polygenic regressions, which can arise in genetically heterogeneous populations (Mills et al. 2020).

Recently, the WLS released a set of polygenic scores (PGSs) (Benjamin et al. 2021), derived from multiple genome‐wide association studies (GWAS), for both the original graduates and their siblings. These PGSs are based on summary statistics from three sources: GWAS conducted by 23andMe, the UK Biobank, and other published studies. The scores were computed using LDpred on HapMap3 SNPs. Only PGSs with an out‐of‐sample predictive power exceeding 1% were included. When multivariate PGSs (estimated using MTAG; Turley et al. 2018) were available, we used those; otherwise, univariate PGSs were used (Benjamin et al. 2021). Detailed documentation is available at https://researchers.wls.wisc.edu/data/polygenic‐scores/. Data and polygenic scores can be downloaded at: https://researchers.wls.wisc.edu/data/polygenic‐scores/ The polygenic score delay discounting was estimated on the basis of the GWA study of Sanchez‐Roige et al. (2018). It was estimated that a low value indicates a predisposition for later gratification and a high value for earlier gratification. Accordingly, in terms of life history, a low value represents a “slow life history strategy” and a high value a “fast life history strategy”.

We aimed to explore the association of phenotypic life history traits as well as the genetic disposition of these life history traits on the genetic predisposition of delay discounting (polygenic scores of delay discounting). We included the following life history traits: (i) the reproductive traits age at first birth and number of children and (ii) education, which may enhance individual resources, enduring performance and survival.

Thus, polygenic scores include PGS of education, PGS of age at first birth, PGS of number of children, and the PGS of delay discounting. Corresponding phenotypic variables included years of postsecondary education (measured in 1964, ranging from 12 to 20 years), age at first birth, number of children and, in all models, year of birth, and, to control for population structure, the first 10 genomic principal components (PCs) provided by the WLS (Benjamin et al. 2021).

We estimated the following models separately for men and women:

- **Genetic models**: Regression using separate linear models of the delay discounting PGS regressing on the PGS of education, age at first birth, and number of children, controlling for year of birth and the first 10 PCs.
- **Phenotypic models**: Regression using separate linear models and general linear models of the delay discounting PGS regressing on the phenotypes of years of education, age at first birth, and number of children, again controlling for year of birth and the first 10 PCs.

For the number of children, we used a general model with a Poisson error structure; otherwise, with a normal error structure. For *R* ^2^ of the Poisson model, we calculated Nagelkerke pseudo *R* ^2^.

## 3 Results

### 3.1 Associations Among Polygenic Scores

We found that in both men and women the PGS of education is significantly negatively associated with the PGS of delay discounting, explaining a very large proportion of variance. However, as stated in the introduction, its magnitude should not be interpreted as a quantitative estimate of pleiotropy. Yet the negative direction of the association aligns with previously reported genetic correlations between discounting and educational attainment. Similarly, also for both men and women, the PGS of age at first birth is significantly negatively associated, whereas the PGS of number of births is significantly positively associated with the PGS of delay discounting (Table 1).

**TABLE 1:** Association among polygenic scores.

Regression models	Estimate	Std. error	*t*‐value	*p*	*R* ^2^%
PGS Educ_Women_x_PGS_delay	−0.8985	0.0079	−113.2980	*p* < 0.0001	79.3070
PGS Educ_Men_x_PGS_delay	−0.8955	0.0085	−105.4000	*p* < 0.0001	79.3110
PGS Afb_Women_x_PGS_delay	−0.7636	0.0117	−65.2580	*p* < 0.0001	57.2750
PGS Afb_Men_x_PGS_delay	−0.7573	0.0125	−60.4070	*p* < 0.0001	56.7190
PGS NBirth_Women_x_PGS_delay	0.3636	0.0170	21.4020	*p* < 0.0001	13.0070
PGS NBirth_Men_x_PGS_delay	0.3700	0.0177	20.8650	*p* < 0.0001	13.5180

### 3.2 Phenotypic Associations With PGS Delay Discounting

The phenotypic polygenic score associations explain much less variance, although the estimates suggest comparable associations and directions. Years of education after high school is significantly negatively associated with the PGS of delay discounting in both men and women, this association explaining 4.7% and 4.5% of the variance respectively (Table 2). The same is true for the phenotype age at first birth for both men and women, but it explains only 1.9% (women) and 1.06% (men) of the variance. The association between number of children and delay discounting is positive in both men and women, but only marginally significant in women and explains 2% of the variance in women (Table 2).

**TABLE 2:** Phenotypic associations with polygenic scores delay discounting.

Regression models	Estimate	Std. error	*t*‐value	*p*	*R* ^2^%
Educ_Men_x_PGS_delay	−0.5616	0.0487	−11.5430	*p* < 0.0001	4.7060
Educ Women_x_PGS_delay	−0.4445	0.0378	−11.7630	*p* < 0.0001	4.5030
Afb_Women_x_PGS_delay	−0.1432	0.0200	−7.1650	*p* < 0.0001	1.9218
Afb_Men_x_PGS_delay	−0.1203	0.0213	−5.6420	*p* < 0.0001	1.0593
Num. Child_Men_x_PGS_delay	0.0135	0.0126	1.0730	*p* = 0.2833	0.1000
Num. Child_Women_x_PGS_delay	0.0207	0.0114	1.8230	*p* = 0.06835	2.0000

## 4 Discussion

Our results are in line with predictions derived from life history theory by showing that the genetic associations among delay discounting, education, and reproductive timing align with the expectations of the fast–slow life‐history conceptual framework. Although phenotypic associations between delay discounting, education, and fertility timing may appear obvious, the contribution demonstrates that these relationships also appear at the molecular genetic level. Because environmental covariation can produce similar phenotypic patterns, detecting corresponding genetic associations is not trivial and presumably provides additional insight into the biological architecture of life‐history–related traits.

The found associations reflect a pattern consistent with a “fast” life‐history strategy, characterized by prioritization of immediate reproduction over long‐term investment in personal development such as education. This is in line with both psychological (LHT‐P) and evolutionary biological (LHT‐E) interpretations of life history theory (Nettle and Frankenhuis 2020). Traditionally, LHT‐P has conceptualized life‐history strategies as mainly environmentally calibrated systems, where organisms adjust behavioral traits in response to early‐life conditions. Our results suggest that, alongside environmental inputs, there may be genetically influenced predispositions that shape tendencies of future orientation, impulsivity, and reproductive timing. This does not contradict the psychological framework but extends it by indicating that individuals may vary in how readily they adopt certain life‐history–relevant behaviors. In this sense, the genetic architecture observed here may influence strategies individuals are likely to adopt, whereas environmental factors determine the expression of these strategies. Integrating genetic predispositions into LHT‐P may therefore provide a more complete approach of how fast–slow patterns emerge across individuals.

The observed associations between the PGS for delay discounting and other PGSs—particularly those for education and age at first birth—show very high levels of shared variance, approximately 80% and 55%, respectively. However, as PGS–PGS regressions are influenced by sample overlap and population structure, the magnitudes cannot be interpreted as an estimate of pleiotropy (Kullo et al. 2022). Accordingly, the PGS–PGS results should be viewed as qualitatively consistent with established genetic correlation patterns rather than quantitative measures of shared genetic architecture. Yet, our finding may give a hint to the interconnectedness of impulsivity, cognitive development, and reproductive behavior, but not to the magnitude of connectedness, strengthening the claim that these traits share a common evolutionary and genetic basis.

The phenotypic associations with the PGS of delay discounting explain only a small proportion of variance in phenotypes as it is usually the case (Mills et al. 2020). Accordingly, the polygenic score for delay discounting explains only about 4.5% of the variance in years of education, and 1%–2% for age at first birth and number of children. Nevertheless, the fact that 4.5% of the variance can be explained by cross‐predicting the polygenic score (PGS) for delay discounting is remarkable, particularly when compared with the variance typically accounted for by directly predicting a phenotype using its own PGS (see Table S1 in the supplement). For example, the PGS for education explains approximately 5% of the variance in the years of education after high school. The polygenic scores for age at first birth explain 1.8% of the variance among men and 4.1% among women, and the polygenic score for number of children ever born explains only 0.7% among men and 0.8% among women.

The consistent direction and significance of these phenotypic associations confirm that genetic predispositions do manifest in observable life outcomes, though to a lesser extent than suggested by the genotypic data alone. This also suggests room for “environmental intervention” and of “environmental modulation” in shaping life trajectories.

Sex‐specific analyses reveal some differences in the strength and significance of associations, but no fundamental differences between men and women; thus, differences may reflect sex‐specific biological pathways or varying social norms and roles that influence the phenotypic expression of life‐history strategies.

More broadly, these findings contribute meaningfully to the ongoing debate between life history theory as conceptualized in psychology versus evolutionary biology. The strong genetic correlations observed here support the view that individual behavioral traits, such as delay discounting, are evolutionarily rooted, possessing a substantial genetic basis. Life history traits are often described mainly from an environmental perspective, but they seem to have a much stronger genetic basis as often suggested. This lends credibility to a more integrated framework that combines insights from both LHT‐P and LHT‐E, genetics and evolution (Nettle and Frankenhuis 2020).

However, the study has limitations that should be acknowledged. Most notably, the phenotypic effects, whereas significant, are modest in size, underscoring the crucial role of environmental influences. Additionally, the genetic data are derived from individuals of European ancestry, limiting the generalizability of the findings across populations. Future research should aim to replicate these results in more diverse samples and incorporate longitudinal designs to track developmental trajectories over time. Expanding the range of traits studied—such as incorporating hormonal profiles, neurobiological measures, or epigenetic markers—could further elucidate the mechanisms linking delay discounting to broader life‐history patterns. Finally, Wisconsin Longitudinal does not provide any phenotypic measure for delay discounting.

Part of our phenotypic associations may reflect indirect genetic effects via parental environments. However, as parental PGS are not included in our data, we cannot control for parental PGS. Future work could also incorporate parental genotypes to separate direct genetic effects from indirect parental genetic influences (genetic nurture), for example using approaches illustrated in Alajääskö et al. (2025).

## 5 Conclusion

In summary, our findings indicate that delay discounting, whereas primarily a behavioral marker of impulsivity, also shows directionally consistent genetic associations with key life‐history–related traits such as educational attainment, reproductive timing, and number of children. These genetic patterns align with expectations from fast–slow life‐history frameworks, suggesting that the predisposition to discount future rewards may form part of a broader constellation of traits involved in long‐term investment and reproductive strategies. Although the corresponding phenotypic associations are modest, their consistency with the genetic results highlights the joint influence of biological and environmental factors on human developmental trajectories. By bringing together perspectives from psychology, evolutionary biology, and statistical genetics, this study suggests that delay discounting may serve as a useful connecting construct for understanding how individual behavioral tendencies relate to life‐history–relevant outcomes, whereas recognizing that these associations do not by themselves validate any specific evolutionary model.

## Funding

The authors have nothing to report.

## Ethics Statement

This study uses secondary data obtained from the Wisconsin Longitudinal Study (WLS), administered by the University of Wisconsin–Madison. All ethical considerations related to participant recruitment, data collection, and consent were handled entirely by the WLS research team. The WLS operates under the oversight of the University of Wisconsin–Madison Institutional Review Board (IRB), and all data were collected in accordance with approved ethical protocols.

## Conflicts of Interest

The authors declare no conflicts of interest.

## Supporting information


**Table S1:** Prediction of phenotypes by it's polygenic scores.

## Data Availability

The data that support the findings of this study are available in Wisconsin Longitudenal Study at https://wls.wisc.edu/.
