# Supplementary material for: Genetic and Phenotypic Associations of the Polygenic Score of Delay Discounting and Life History Traits
Source: Am J Hum Biol. 2026 Jan 7;38(1):e70192. doi: 10.1002/ajhb.70192 (PMC12780642; doi:10.1002/ajhb.70192)
Supplement: Supplementary file 1 — Table S1: Prediction of phenotypes by it's polygenic scores. [file AJHB-38-e70192-s001.docx]

**Supplementary Table**

| **Models** | **Estimate** | **Std_Error** | **t_value** | **P** | **R^2^ %** |
| --- | --- | --- | --- | --- | --- |
| Educ_Men_x_PGS_Educ | 0.23912 | 0.019 | 12.585 | P < 0.0001 | 5.55 |
| Educ_Women_x_PGS_Educ | 0.22839 | 0.01838 | 12.427 | P < 0.0001 | 5 |
| Afb_Men_x_PGS_PGS_Afb | 0.14996 | 0.02119 | 7.076 | P < 0.0001 | 1.8 |
| Afb_Women_x_PGS_Afb | 0.21015 | 0.01988 | 10.57 | P < 0.0001 | 4.1 |
| Num._Child_Men_x_PGS_delay | 0.051591 | 0.01261 | 4.091 | P < 0.0001 | 0.7 |
| NumChild_Women_x_PGS_delay | 0.05203 | 0.01138 | 4.572 | P < 0.0001 | 0.8 |

Table S1) Prediction of phenotypes by it’s polygenic scores.
